# Management of first‐time patellar dislocation: The ESSKA 2024 formal consensus—Part 2

**DOI:** 10.1002/ksa.12637

**Published:** 2025-03-07

**Authors:** Peter Balcarek, Lars Blønd, Philippe Beaufils, Marie Askenberger, Joanna M. Stephen, Ramazan Akmeşe, Rene El Attal, Vasileios Chouliaras, Paolo Ferrua, Joan Minguell Monart, Geert Pagenstert, Petri Sillanpää, Manuel Vieira Da Silva, Florian Dirisamer, Jacek Walawski

**Affiliations:** ^1^ Department of Orthopaedics and Traumatology Arcus Klinik Pforzheim Germany; ^2^ Orthopedic Department Zealand University Hospital, Køge and Aleris Hospital Copenhagen Denmark; ^3^ Centre Médical, Fondation Norbert Metz Luxembourg; ^4^ Section of Pediatric Orthopaedic Surgery, Karolinska University Hospital in Solna Stockholm Sweden; ^5^ Department of Mechanical Engineering Imperial College London London England; ^6^ Orthopaedics and Traumatology Department Çankaya Hospital Ankara Çankaya Turkey; ^7^ Orthopedics and Trauma Department Academic Teaching Hospital Feldkirch Austria; ^8^ Orthopaedic Department General Hospital of Arta Arta Greece; ^9^ Dipartimento di Scienze Biomediche per la Salute SCIBIS Università degli Studi di Milano Milan Italy and I Clinica Ortopedica, ASST Gaetano Pini, CTO Milan Italy; ^10^ Orthopaedic Department Vall d'Hebron University Hospital Barcelona Spain; ^11^ CLARAHOF Clinic of Orthopaedic Surgery University of Basel Basel Switzerland; ^12^ Department of Orthopedics and Trauma Pihlajalinna Hospital Tampere Finland; ^13^ Orthopedic Department Trofa Saúde Hospital Braga Centro Braga Portugal; ^14^ Praxis Move Puchenau Austria; ^15^ Orthopedic Department ŻagielMed Hospital, MSWiA Hospital Lublin Lublin Poland

**Keywords:** first‐time patellar dislocation, formal consensus, MPFL, osteochondral lesion, outcome, patellar instability

## Abstract

**Purpose:**

To provide recommendations for the treatment of patients with first‐time patellar dislocation (FTPD). Part 2 focused on nonoperative treatment, bracing, rehabilitation, indications for surgery and surgical strategies.

**Methods:**

The consensus was performed according to the European Society for Sports Traumatology, Knee Surgery and Arthroscopy consensus methodology.

**Results:**

The consensus comprised 32 questions and statements, 19 of which will be presented in this part. Eight statements achieved strong agreement (median 9; range 7–9), and 11 statements achieved relative agreement (median 9; range 5–9). None were Grade A, 2 were Grade B, 11 were Grade C and 5 were Grade D. In summary, treatment decisions for FTPD should prioritize individualized care, balancing patient‐specific risks and demands. Surgical options are increasingly considered for skeletally immature patients and those with increased recurrence risk. Medial patellofemoral ligament (MPFL) reconstruction is the preferred surgical technique for addressing medial soft tissue stabilizers, offering better outcomes than repair methods. Combining MPFL reconstruction with corrections of relevant bony risk factors might further reduce the risk of recurrence and revision surgery, although specific thresholds for intervention remain debated. Physical therapy is recommended as an essential complement to both operative and nonoperative treatments, but bracing offers no clear long‐term benefit. Chondral or osteochondral lesions should be repaired when the defect is at least 1 cm² in the patellofemoral joint contact area. Fragment refixation or other cartilage restoration techniques are preferred, and delayed repair is favoured over fragment removal when immediate surgery is not needed.

**Conclusion:**

The consensus consists of recommendations for evaluation and treatment strategies for managing FTPD. High levels of agreement were reached by experts throughout Europe. In areas without clear scientific evidence, this consensus aimed at providing recommendations and guidance on the basis of expert opinion and pointed out areas where further studies are necessary.

**Level of Evidence:**

Level I, consensus.

**Abbreviations**
ESSKA: European Society for Sports Traumatology, Knee Surgery and Arthroscopy
FTPD: first‐time patellar dislocation
MPFL: medial patellofemoral ligament
MPFL‐R: medial patellofemoral ligament reconstruction
MPTL: medial patellotibial ligament
OCF: osteochondral fragment
PROM: patient‐reported outcome measure

## INTRODUCTION

The management of first‐time patellar dislocation (FTPD) involves significant debate [31, 52]. Nonoperative (conservative) management has traditionally been recommended for patients experiencing FTPD without significant osteochondral lesions [2, 8, 35, 38, 53]. However, high redislocation rates that increase with longer follow‐up periods are striking, and emerging evidence supports individualized management strategies to reduce recurrent instability events and improve patient‐reported outcomes in both skeletally mature and immature patients [9, 15, 30, 36, 43].

Recent trends in managing FTPD, in those with and without intraarticular fractures or loose bodies, highlight a shift toward early surgical intervention [9, 24, 30]. Primary reconstruction of the medial patellofemoral ligament (MPFL‐R) is increasingly recommended to prevent further dislocation and associated cartilage damage [17, 22]. This approach reflects a growing recognition of the limitations of conservative treatment [33]. However, the current literature is still inconclusive regarding the indication and need for concomitant bony correction in addition to MPFL‐R [6]. In particular, in skeletal immature patients, the postoperative functional outcome scores of MPFL‐R are not necessarily superior to those of other soft‐tissue techniques [55].

Several studies have aimed to identify certain risk factors crucial for patient‐reported outcomes and patellar stability; the significance of these risk factors relevantly varies among studies [10]. Given the natural heterogeneity in patients with FTPD and inconsistencies in reporting risk factors, many published articles have limited applicability when reporting results after isolated MPFL‐R [52]. In addition, the lack of definitions for significant bony abnormalities and threshold values affecting the outcome of treatment for FTPD remains a challenge.

The available literature on FTPD management is characterized by considerable heterogeneity in demographics, risk factors, surgical techniques, and outcome measures. Many studies fail to distinguish between FTPD and recurrent dislocations or between skeletally immature and mature patients. These inconsistencies limit the generalizability of findings and underscore the need for more rigorous, stratified research. The objectives of this consensus are to provide recommendations and guidance for the treatment of patients with FTPD based on evidence from the literature and clinical expertise of a well‐distributed panel. Part 2 presents a synopsis of the entire Consensus in condensed form, focusing on nonoperative treatment, bracing, rehabilitation, indications for surgery and surgical strategies for managing FTPD. The full text with the complete reference list can be accessed at https://esskaeducation.org/esska-consensus-projects and https://esskaeducation.org/webcasts/management-first-time-patella-dislocation-ftpd-esska-formal.

## MATERIALS AND METHODS

The Patellofemoral Instability Committee of the European Society for Sports Traumatology, Knee Surgery and Arthroscopy (ESSKA) first initiated the FTPD consensus process in April 2022. The method was based on the ‘formal consensus process’ as described by the French National Healthcare Institution (Haute Autorité de Santé HAS) and was specifically published by ESSKA [5, 6, 18]. A total of 55 European surgeons and scientists were involved in the process, comprising steering (13 orthopaedic surgeons, 1 physiotherapist), rating (24 experts) and peer‐review groups (17 orthopaedic surgeons). The two principal objectives of the Steering Committee were to (A) devise a framework of questions suitable for consensus and educational purposes and (B) thoroughly evaluate the scientific literature and combine it with expert opinion to produce robust statements (please see also Part 1).

### Question group

The question group comprised three experts who formulated a series of enquiries to cover the relevant and important aspects of FTPD.

### Literature group

The literature group, comprising the remaining members of the steering group, performed a literature search with the endpoint by 31 May 2022, including PubMed, Google Scholar and EMBASE, according to keywords relevant to each specific question. The title and abstract of each reference were evaluated, and any relevant article was then obtained in full for the steering group to summarize as a brief report. Peer‐reviewed clinical studies with levels of evidence ranging from 1 to 5 were included in this analysis.

### Number of question‐statements

In total, 32 questions and accompanying statements were formulated and presented to the rating group. Part 1 focused on clinical presentation, symptoms, diagnosis, evaluation, imaging and complications (questions 1–9 and 27–29). Part 2 focused on nonoperative treatment, bracing, rehabilitation, indications for surgery and considerations of surgical strategies (questions 10–26). Each statement was designated on the basis of its grade of recommendation, that is, Grades A, B, C or D. A—high scientific level, B—scientific presumption, C—low scientific level and D—expert opinion.

### Rating group

The statements were rated by a group of 24 patellofemoral experts across European countries. Each expert was asked to evaluate all questions and statements via a 1‐ to 9‐point Likert grading scale. Values of 2–8 represented possible intermediate situations. A proposal was deemed appropriate when the value of the median was ≥7 and the score of each rater was ≥5, indicating relative agreement, and strong agreement when the value of the median was ≥7, with no singular rater score <7. The results are shown as the median and range.

### Peer‐review group

The peer‐review group was composed of 17 European surgeons selected from ESSKA‐affiliated European national arthroscopic and/or sports and trauma societies, who routinely manage patients with patellofemoral instabilities. The review group was specifically required to evaluate the recommendations in the final version for their relevance to a diverse European readership, together with their geographical adaptability and readability. The comments were incorporated into the final version.

## RESULTS

In the first round, the 32 questions were presented to the rating group, and all the questions were accepted; thus, no questions were deleted, and no questions were added. Twelve of the 32 questions and statements needed revision after the first round and had to be rated again in the second round. Six of the 12 questions belong to Part 2. Nineteen questions and statements will be presented in this part. Eight statements achieved strong agreement (median 9; range 7–9), and 11 statements achieved relative agreement (median 9; range 5–9). None were Grade A, 2 were Grade B, 11 were Grade C and 5 were Grade D.

**(10) Is nonoperative treatment the gold standard for treating FTPD in skeletally mature patients?**

In skeletally mature patients, conservative treatment was the standard and most common treatment for FTPD in the past. Today, with respect to recent literature, there is a need to assess predisposing factors and the risk of ongoing symptoms and recurrence to make the final decision. Treatment should also be tailored to patient characteristics and demands. Therefore, the consensus group suggests conservative treatment only in patients with low recurrence risk (see also question 14) and without chondral or osteochondral lesions.

**Grade**: C

**Rating:** median 9 (range 5–9). Relative agreement

**(11) Is nonoperative treatment the gold standard for treating FTPD in skeletally immature patients?**

Given the high recurrence rates in children, we cannot support nonoperative treatment as the gold standard for every patient. Skeletally immature patients with FTPD need to be thoroughly investigated to clarify the extent of predisposing factors. Individual redislocation risk estimation and a check for osteochondral or chondral fragments should be performed to stratify between surgical or nonsurgical treatment and extensively discuss with the patient and family. If the decision for nonoperative treatment is met, strict follow‐up is mandatory.

**Grade**: C

**Rating:** median 9 (range 6–9). Relative agreement

**(12) What is the role of bracing for nonoperative treatment in FTPD?**

There is no evidence for the superiority in the use of any brace (as opposed to no bracing) in FTPD either in the acute or in the nonacute phase. Bracing with an unrestricted range of motion may only be considered in the acute phase after FTPD for a very short time.

**Grade**: B

**Rating:** median 9 (range 5–9). Relative agreement

**(13) Is there any evidence for physical therapy in FTPD patients?**

Despite the lack of strong scientific evidence for the benefit of physical therapy in patients with FTPD, the consensus group recommends physical therapy‐guided exercises as a necessary supplement in operative or nonoperative treatment. Physical therapy in FPTD patients is focused not only on quadriceps and gluteal muscle strengthening but also on motion exercises, gait re‐education, functional neuromuscular control and sport‐specific training where indicated.

**Grade:** C

**Rating:** 9 range (5–9). Relative agreement

**(14) In which cases should surgical treatment be considered in skeletally mature patients?**

In skeletally mature patients, the individual analysis of risk factors and the estimation of recurrence risk using one of the published scoring systems and/or ongoing symptoms or osteochondral lesions should be the basis for decision‐making. Therefore, primary surgical reconstruction needs to be considered a first option in patients with several risk factors and ongoing symptoms or osteochondral lesions, ultimately changing the old paradigm of first‐line nonoperative treatment in all patients. The pros and cons of surgical versus nonsurgical treatment need to be carefully discussed with the patient.

**Grade:** C

**Rating:** median 9 (range 6–9). Relative agreement

**(15) In which cases should surgical treatment be considered in skeletally immature patients? Are there any specific clinical concerns in skeletally immature patients after FTPD?**

There is a greater incidence of persistent symptoms after FTPD, especially among skeletally immature patients than among skeletally mature patients. Therefore, the risk for further instability and thereby a greater risk of cartilage deterioration and loss of knee‐related quality of life are substantial among adolescents. Current individualized (á la carte) surgical techniques demonstrate good outcomes in patients with open growth plates. Due to high recurrence rates and age itself being a risk factor, surgical treatment should be discussed with patients and families for every skeletally immature patient. The decision should be made respecting the individual risk for instability, ongoing symptoms and/or osteochondral fragments (OCFs) and patient demands.

**Grade:** C

**Rating:** median 9 (range 5–9). Relative agreement

**(16) Is there a role for medial patellofemoral ligament (MPFL)/medial patellotibial ligament (MPTL) repair versus reconstruction in acute cases?**

MPFL reconstruction has demonstrated clinical superiority compared to medial soft tissue repair techniques (MPFL/MPTL repair). Therefore, when MPFL surgery is indicated for patients with FTPD, MPFL reconstruction is the method of choice to address the medial soft tissue stabilizers.

**Grade**: C

**Rating:** median 9 (range 6–9). Relative agreement

**(17) Has MPFL reconstruction a better outcome than MPFL repair?**

Comparative studies are very heterogeneous in terms of demographics, risk factors and surgical techniques and are not limited to patients with FTPD. With today's knowledge of individualized treatment regarding risk factors and skeletal maturity taken into consideration, MPFL reconstruction has a better outcome than MPFL repair.

**Grade:** C

**Rating:** median 9 (range 6–9). Relative agreement

**(18a) When is isolated MPFL reconstruction indicated in FTPD?**

**(18b) Should combined corrective surgery (MPFL + additional procedure) be considered for treating FTPD in the presence of (bony) anatomic abnormalities?**

Despite the lack of clear scientific evidence supporting these questions, the consensus group supports isolated MPFL reconstruction in the absence of relevant bony risk factors and, vice versa, the correction of relevant bony risk factors in addition to MPFL reconstruction after FTPD. As was shown for redislocations, untreated bony risk factors increase the risk of failure. For this reason, it is only consistent to correct relevant bony abnormalities at the earliest possible chance to avoid recurrence and revision surgery. The exact cutoff for relevant bony abnormalities is under debate, and therefore decision is always individual.

**Grade:** D

**Rating:** median 9 (range 7–9). Strong agreement

**(19) In case combined procedures are necessary, should these be done simultaneously or staged?**

Despite a lack of literature data, the consensus group supports the single‐stage correction of relevant instability factors whenever needed and possible. In selected very complex cases (such as derotation osteotomies), a staged procedure may be an option. Uncorrected risk factors may have a negative influence not only in terms of recurrence or knee‐related quality of life but also deteriorate the result of already addressed reconstructions. There are no clear cut‐off values available to indicate these combined procedures.

**Grade:** D

**Rating:** median 9 (range 8–9). Strong agreement

**(20a) Which osteochondral or chondral fragments need surgery?**

To achieve the highest possible degree of knee restoration, at least osteochondral or chondral fractures should be repaired if the defect is in the patellofemoral articular area and equal to or larger than 1 cm^2^. Depending on the flake characteristics, one can consider fragment refixation or other cartilage restoration techniques.

**Grade:** C

**Rating:** median 9 (range 7–9). Strong agreement

**(20b) Which FTPD patients need immediate surgery? When should delayed surgery be considered?**

Even when repairing a chondral or osteochondral lesion at the earliest possible time point is preferable, delayed refixation or repair rather than fragment removal should be considered. The patient workup (especially imaging) should ideally be performed before this acute surgery to understand the risk factors and to have the option to add concomitant procedures if necessary. In the absence of repairable chondral or osteochondral injuries, immediate surgery is not necessary.

**Grade:** D

**Rating:** median 9 (range 7–9). Strong agreement

**(21) Are there any differences between skeletal mature and immature patients who should be treated for chondral or OCFs?**

Refixation of chondral or OCFs should be the first line of treatment. In general, skeletal immaturity should not make any difference when treating chondral or osteochondral defects after FTPD but should have a lower threshold for repairing even smaller defects.

**Grade:** D

**Rating:** median 9 (range 7–9). Strong agreement

**(22) Is there an indication for concomitant MPFL reconstruction when surgery is performed for osteochondral/chondral injuries or loose bodies in FTPD in children and adolescent patients or skeletally mature patients?**

When a chondral or osteochondral fracture has to be surgically treated, a concomitant stabilizing procedure should be used. MPFL reconstruction is the recommended procedure for addressing injured medial structures. Skeletal immaturity should not influence this indication but may require a specific surgical technique.

**Grade:** C

**Rating:** median 9 (range 6–9). Relative agreement

**(23) How can malalignment and patellofemoral bony abnormalities be addressed in open physes?**

At the same time, open physes are both chance and problem (see also question 24). Guided growth to correct coronal malalignment is a method with high potential that is much easier than osteotomy in adults and should be considered when necessary. Axial malalignment can be corrected without harming the physes. Therefore, this can be carefully considered with respect to the dynamic torsion values during growth. Other bony techniques addressing the tibial tubercule or the trochlear geometry are generally not recommended for wide‐open physes; specific paediatric techniques can be used in selected cases. There are soft tissue options to address patella alta or a lateralized tibial tubercule if needed. Trochleoplasty can be performed safely close to skeletal maturity.

**Grade:** C

**Rating:** median 8.5 (range 5–9). Relative agreement

**(24) Is there an indication for growth‐directing procedures (e.g., hemiepiphyseodesis) in patients with open growth plates?**

The presence of valgus knees is one of the anatomical risk factors for patellar instability and can be a major risk factor for some children. Even less valgus of 3–5° in a growing child with patellar instability should be considered for correction. Alignment is part of the clinical examination and can be further visualized with radiographs and long‐leg standing X‐rays. When treating children, there is a possibility of minor surgery to correct the valgus deformity when there is remaining growth prior to or at the same time as the MPFL reconstruction.

**Grade:** D

**Rating:** median 8 (range 5–9). Relative agreement

**(25) What is the redislocation rate after conservative treatment of FTPD?**

The recurrence rate after conservative treatment for FTPD is at least 25%, and the percentage of patients with persistent symptoms in addition to recurrent dislocation is approximately 50%. The highest recurrence rates can be expected in children and adolescents (up to 70%).

**Grade:** B

**Rating:** median 9 (range 9–9). Strong agreement

**(26) What is the recurrence rate after surgical treatment of FTPD?**

With the correct evaluation of patients and correct surgical techniques, the outcome of surgical intervention is superior to that of conservative treatment. Some of the literature is based on data on outdated procedures such as MPFL repair or medial reefing, procedures known to demonstrate recurrence rates similar to those of conservative treatment. Few studies have focused on outcomes after MPFL reconstruction in patients with FTPD, but the current knowledge strongly indicates that the recurrence rate is lower in these patients than in those receiving conservative treatment.

**Grade:** C

**Rating:** median 9 (range 8–9). Strong agreement.

## DISCUSSION

The most important findings of this consensus are that treatment decisions for FTPD should prioritize individualized care, balancing patient‐specific risks and demands. Surgical options are increasingly considered for skeletally immature patients and those with increased recurrence risk, shifting treatment to a more patient‐centred strategy. MPFL‐R is the preferred surgical technique for addressing medial soft tissue stabilizers. Combining MPFL‐R with corrections of relevant bony risk factors might further reduce the risk of recurrence and revision surgery accompanied by improved clinical outcomes, although specific thresholds for intervention remain debated. Physical therapy is recommended as an essential complement to both operative and nonoperative treatments, but bracing offers no clear long‐term benefit. Chondral or osteochondral lesions should be repaired when the defect is at least 1 cm^2^ in the patellofemoral joint contact area. Fragment refixation or other cartilage restoration techniques are preferred. For both scenarios, a concomitant MPFL‐R is recommended.

The incidence of FTPD and its recurrence are highly variable, depending on the selected patient groups and administrative data sets used [14, 21, 44], and tend to be the highest among active adolescents and athletic cohorts [16]. Every FTPD has its own characteristics regarding the injury mechanism, injury pattern and presence of anatomic patellar instability risk factors, including an inherent risk of further dislocating events, cartilage lesions and osteoarthritis in the long term [3, 45, 51, 54]. As the number of factors present in a single patient increases, the risk of experiencing recurrent dislocation also increases [1, 4, 19]. Although sex and the MPFL injury pattern are nonsignificant factors for experiencing patellar redislocation, young age/skeletally immature status, trochlear dysplasia and patella alta were found to be significant risk factors in nearly all risk stratification models considering patellar instability recurrence [1, 4, 19, 27, 29].

Nonoperative management has often been considered the first‐line treatment for patellar instability despite the high rate of redislocation, which increases with follow‐up (71% at 14 years) [38]. However, more recent evidence suggests individualized treatment on the basis of predisposing factors and that stratifying risk favours surgery [7, 9, 15]. In 2019, Pagliazzi et al. published a meta‐analysis of surgical versus nonsurgical treatment of FTPD and confirmed a greater redislocation rate with the nonsurgical approach and more favourable clinical outcomes with the surgical approach during short‐ and midterm follow‐up (6 years) [37]. Focusing on MPFL reconstruction techniques in recurrent dislocations, Cohen et al. reported redislocation rates of only 7% in the operatively treated group versus 30% in the rehabilitation group [9]. This was accompanied by a higher Kujala score in the surgically treated group (81 pts. vs. 87 pts.), but there were no differences in patellofemoral pain between the groups. These findings are in line with another systematic review of 12 studies that reported significantly improved Kujala scores after MPFL‐R compared with nonoperative treatment in patients with both FTPD and recurrent dislocations [30]. Thus, in patients with more than one dislocation, the MPFL‐R increasingly showed clear advantages in terms of the redislocation rate and functional outcome compared with those of other soft‐tissue procedures, at least when the Kujala score was used as the outcome parameter [22, 30, 41]. Other patient‐reported outcome measures (PROMs; e.g., International Knee Documenting Committee and Tegner score) are not as clear in this regard. Similar results have also been reported in the treatment of patients with FTPD, despite the use of smaller data sets [24]. Surgical treatment of FTPD in children and adolescents was also associated with a lower risk of recurrent dislocation [12, 36] and, in some investigations, was associated with higher health‐related quality of life and sporting function considering various surgical treatment procedures [36]. The MPFL‐R appears to be beneficial in skeletally immature patients with redislocation rates comparable to those of adults [11, 47]. However, there is a paucity of evidence on MPFL‐R for FTPD in the skeletally immature population in that the postoperative functional outcome scores of MPFL‐R are not necessarily superior to those of other soft‐tissue techniques [55]. In addition, in children and adolescents, Wilkens et al. reported no statistically significant differences in recurrent instability rates between the MPFL‐R technique and other soft tissue realignment techniques [55].

The incidence of patellar dislocation complicated with OCFs, as well as the recurrence rate of instability, is high in the paediatric and adolescent age groups. The presence of an OCF, the extent of primary cartilage damage, and the number of further dislocation events significantly impact the process of early joint degeneration [26, 45, 46]. Therefore, chondral or osteochondral lesions should be repaired when the defect is at least 1 cm² in the patellofemoral joint contact area. Fragment refixation or other cartilage restoration techniques are preferred, and delayed repair is favoured over fragment removal when immediate surgery is not required in both skeletally mature and immature patients. Even if early treatment makes successful healing more likely, the time interval between injury and operation seems to be less important, and fragment refixation should be attempted even in a nonacute setting if the size and integrity of the OCF allow for stable fixation [13, 23].

Gurusamy et al. reported that patients who simultaneously underwent MPFL‐R had significantly lower redislocation rates than those who underwent MPFL repair or no additional MPFL treatment (10% vs. 58.7%) [17]. In addition, studies that included MPFL‐R in the treatment of FTPD complicated by articular fragments have shown low rates of patellar redislocation [28, 42]. Thus, when a chondral or osteochondral fracture has to be surgically treated, a concomitant MPFL‐R is the recommended procedure. Skeletal immaturity should not influence this indication but may require a specific surgical technique not violating the open physis. To address the most common risk factors, additional surgical procedures that alter the bony anatomy of the patellofemoral joint have been introduced (i.e., tibial tubercle osteotomy, trochleoplasty, and varus‐producing/derotational osteotomy). Most bony procedures are believed to be necessary in the case of severe bony abnormalities [56, 57, 58]. To date, the literature does not support any specific threshold values for these abnormalities, regardless of whether surgery is warranted, although these abnormalities may play a role in individual dislocation prognosis [1, 4, 19, 27, 29]. In particular, whether bony abnormalities should be addressed in FTPD to minimize the risk of redislocation has not been studied. However, the consensus group supports the correction of relevant bony risk factors (one‐step procedure or staged) in addition to MPFL‐R after FTPD since uncorrected risk factors may have a negative influence not only in terms of recurrence or knee‐related quality of life but also on the results of previously reported reconstructions.

In skeletally immature patients, bony procedures may be contraindicated because of the open distal femoral and proximal tibial physis and the risk of growth disturbance if the physeal region is exposed to surgery. However, specific surgical techniques have been described to be utilized in skeletally immature patients considering MPFL‐R [48], addressing patella alta [40] and correction of lower limb malalignment in the coronal plane by hemi‐epiphysiodesis, either at the distal femur or proximal tibia [39]. The current literature considering growth guidance for patellar instability associated with genu valgum is limited to a few case series of patients with recurrent dislocations. Thus, a clear recommendation on growth modulation after FTPD can hardly be made. Nevertheless, it seems reasonable to perform selective hemi‐epiphysiodesis in patients whose constellation after FTPD requires primary surgical treatment and whose genu valgum is a relevant anatomical risk factor for instability. However, the current literature lacks, similar to skeletally mature patients, any scientific evidence on whether bony risk factors should be addressed in FTPD and which technique should be used.

Physical therapy is widely used in the management of FTPD despite a lack of supporting evidence. A recent systematic review revealed that despite high mean PROM scores following physical therapy intervention in FTPD patients, preinjury knee function is not restored [33]. However, physical therapy for FTPD patients should help address deficiencies likely to increase knee valgus and rotation (e.g., excessive femoral adduction and internal rotation, tibial rotation, and foot pronation) since this is a known risk factor for patellar dislocation [34]. Quadriceps strengthening is the most widely investigated intervention with the highest‐level evidence, but there is no clear consensus on the definition of optimal rehabilitation following FTPD [33, 49]. Management typically includes range of motion exercises, gait re‐education, techniques to promote soft tissue flexibility, functional neuromuscular control, strengthening exercises and sport‐specific interventions [32], but the role of physical therapy in FTPD needs further investigation since there is currently limited evidence to support its superiority to leaving patients to natural progression [31].

Like physical therapy, there is no consensus about the type and duration of immobilization, but most studies use some form and variable time of bracing [25, 50]. The different modalities can be roughly classified into two groups. One is 6 weeks of immobilization using a cylinder cast or removable splint to allow healing of the medial retinaculum and soft tissues, encouraging fibrosis and the repair of stretched or ruptured stabilizing tissues. The other is functional mobilization after a short immobilization period in which a simple brace is used to avoid the detrimental effects of muscle atrophy, thereby accelerating recovery. The use of elastic bracing results in dislocation rates similar to those of more restrictive immobilization but shows the benefits of reducing associated muscle hypotrophy and improving the range of motion of the knee [20]. However, further high‐level studies with modern braces and standardization of immobilization time and controlling variables, including age and predisposing risk factors, are needed to confirm the most appropriate bracing protocol for FTPD patients.

The consensus process, starting from the available evidence and based on expert recommendations, identified scenarios where the management of FTPD is evolving from a one‐size‐fits‐all approach to a more nuanced strategy that considers individual risk factors and patient characteristics and demands. While nonoperative treatments remain viable for patients with minimal risk factors, surgical interventions, particularly MPFL‐R, offer significant advantages for reducing redislocation rates and improving functional outcomes in selected patients. However, the high methodological quality of the consensus process cannot compensate for the limitations of the available evidence. Further research is needed to refine risk stratification models, evaluate the role of physical therapy and bracing, and determine the optimal treatment strategy for addressing bony abnormalities in both skeletally mature and immature patients. Another limitation could be represented by the panel composition, which included many operative clinicians experienced in treating patellar instability, and may consequently be biased in favour of surgical treatment. The authors strongly recommend reading the entire consensus paper, including a more thorough overview of the published literature and the complete list of references (https://esskaeducation.org/esska-consensus-projects and https://esskaeducation.org/webcasts/management-first-time-patella-dislocation-ftpd-esska-formal).

## CONCLUSION

The consensus consists of recommendations for general and specialized orthopedic surgeons for evaluation and treatment strategies for managing FTPD. High levels of agreement were reached by experts throughout Europe. In areas without clear scientific evidence, this consensus aimed at providing recommendations and guidance on the basis of expert opinion and pointed out areas where further studies are necessary and advisable.

## AUTHOR CONTRIBUTIONS

All authors (with the exception of Philippe Beaufils) are members of the steering group and therefore responsible for the conception and implementation of the consensus process. This included the formulation of the questions, the literature research, the summary of the literature and the formulation of the statements. Peter Balcarek and Jacek Walawski wrote the paper, which was reviewed and finally approved by all authors. Philippe Beaufils served as the ESSKA Consensus Projects Advisor.

## CONFLICTS OF INTEREST STATEMENT

Peter Balcarek reports consulting for Arthrex. Philippe Beaufils serves as the ESSKA Consensus Projects Advisor. Florian Dirisamer reports consulting for Arthrex and receives royalties from Arthrex Inc. Rene El Attal reports consulting for Arthrex, DepuySynthes and ZimmerBiomet. Geert Pagenstert reports consulting for DepuySynthes and Stryker. Joan Minguell reports consulting for Arthrex and Smith&Nephew. Petri Sillanpaa reports consulting for Inion LTD. Ramazan Akmeşe reports consulting for Smith&Nephew, and Jacek Walawski reports consulting for Arthrex, Moximed and Smith&Nephew. The remaining authors declare no conflicts of interest.

## ETHICS STATEMENT

This article does not contain any studies with human participants or animals performed by any of the authors.

## Data Availability

The full text with the complete reference list can be accessed at https://esskaeducation.org/esska-consensus-projects and https://esskaeducation.org/webcasts/management-first-time-patella-dislocation-ftpd-esska-formal.
